# How Lonely are Older Americans Act National Family Caregiver Support Program Participants?

**DOI:** 10.1093/geroni/igab046.3403

**Published:** 2021-12-17

**Authors:** Claire Pendergrast, Heather Menne

**Affiliations:** 1 Syracuse University, Syracuse, New York, United States; 2 RTI, RTI, District of Columbia, United States

## Abstract

Older Americans Act (OAA) family caregiver services connect family members caring for older adults with a diversity of community-based resources and supports. Social isolation and loneliness are known public health threats, and family caregivers may face greater vulnerability to loneliness given the often-intensive time demands of care provision. Policy stakeholders and aging services providers are increasingly focused on combating loneliness among older adults and family caregivers. To inform efforts to reduce loneliness, we conducted descriptive analyses to identify characteristics of the participants in the OAA National Family Caregiver Support Program associated with higher rates of loneliness, measured with the 3-item UCLA Loneliness Scale. Using data from the 2019 National Survey of Older Americans Act Participants, we examined how caregiver loneliness varied based on caregiver age, gender, income, race and ethnicity, living alone, rurality, and self-reported health, as well as care recipients’ health status and difficulties with ADLs. Among our sample of 1,824 family caregivers, rates of loneliness were high overall (70%). Loneliness was significantly higher for caregivers with poor health (71.4%), incomes less than $20,000 (75.3%), living alone (75.4%), aged 65 or older (73.2%), Hispanic caregivers (82.2%) and caregivers for care recipients with 3 or more ADLs (76.0%). Findings underscore the importance of increasing social engagement opportunities for family caregivers. Policies and programs focused on reducing caregiver loneliness should be accessible to all family caregivers but should prioritize outreach and engagement for groups at higher risk of loneliness.

